# The Roles of Hormone Signals Involved in Rhizosphere Pressure Response Induce Corm Expansion in *Sagittaria trifolia*

**DOI:** 10.3390/ijms241512345

**Published:** 2023-08-02

**Authors:** Enjiao Li, Jing Tang, Jiexia Liu, Zhiping Zhang, Bing Hua, Jiezeng Jiang, Minmin Miao

**Affiliations:** 1 College of Horticulture and Landscape Architecture, Yangzhou University, Yangzhou 225009, China; 2 Joint International Research Laboratory of Agriculture and Agri-Product Safety, Ministry of Education of China, Yangzhou University, Yangzhou 225009, China; 3 Key Laboratory of Plant Functional Genomics, the Ministry of Education, Yangzhou University, Yangzhou 225009, China

**Keywords:** rhizosphere pressure, plant hormone, corm expansion, transcriptome

## Abstract

Soil is the base for conventional plant growth. The rhizosphere pressure generated from soil compaction shows a dual effect on plant growth in agricultural production. Compacted soil leads to root growth stagnation and causes bending or thickening, thus affecting the growth of aboveground parts of plants. In arrowhead (*Sagittaria trifolia* L.), the corms derived from the expanded tips of underground stolons are its storage organ. We found that the formation of corms was significantly delayed under hydroponic conditions without rhizosphere pressure originating from soil/sand. In the initial stage of corm expansion, the anatomic structure of arrowhead corm-forming parts harvested from hydroponics and sand culture was observed, and we found that the corm expansion was derived from cell enlargement and starch accumulation. Comparative transcriptome analysis indicated that the corm expansion was closely related to the change in endogenous hormone levels. Endogenous abscisic acid and salicylic acid concentrations were significantly increased in sand-cultured corms. Higher ethylene and jasmonic acid contents were also detected in all arrowhead samples, demonstrating that these hormones may play potential roles in the rhizosphere pressure response and corm expansion. The expression of genes participating in hormone signaling could explain the rising accumulation of certain hormones. Our current results draw an extensive model to reveal the potential regulation mechanism of arrowhead corm expansion promoted by rhizosphere pressure, which will provide important references for further studying the molecular mechanism of rhizosphere pressure modulating the development of underground storage organs in other plants.

## 1. Introduction

In agricultural production, soil compaction or mechanical impedance has always been a negative factor for crop growth [1,2]. Early research reported that the growth of plants was weakened [3], the roots became thinner and shallower [4], lateral roots were proliferated [5], the radial growth diameter of roots was increased [6,7], and the shoot growth was inhibited [8] in compacted soil. When plant roots penetrate the soil and extend in all directions, they are subjected to axial mechanical resistance and radial friction. The resistances applied to root growth are summarized as rhizosphere pressure herein. The magnitude of rhizosphere pressure is closely related to the soil’s physical condition, compaction density, and root position [9]. Rhizosphere pressure that roots recognize from different directions has different effects on the root system. According to the research by Feng et al. [10], the positive impedance of soil caused a decreased root length, while lateral pressure promoted the radial enlargement of cortical cells, thus yielding an increased root diameter. Montagu et al. [8] described that the growth of aboveground parts is affected by rhizosphere pressure, whereby the total root length is inhibited. Considerable studies have shown that rhizosphere pressure has a regulatory role in the growth of plant roots and aboveground parts. However, it is still unclear whether rhizosphere pressure has an impact on the development of underground storage organs.

Plant hormones are important regulatory factors in the process of plant growth and development. Several studies have reported that the effects of rhizosphere pressure on underground growth are closely associated with changes in endogenous hormones in plants. Whalen and Feldman [11] found that the phenotypic changes in roots induced by rhizosphere pressure were similar to those induced by exogenous ethylene (ETH). Plant roots produce ETH after sensing rhizosphere pressure, thus promoting the root system to penetrate into the soil [12,13,14]. Additionally, other hormone signals are also involved in mediating the root response to rhizosphere pressure, leading to various phenotypic changes in roots. Tracy et al. [15] put forward the view that the changes in root structure in compacted soil are related to alterations in endogenous abscisic acid (ABA) contents. Moreover, the active bending in the process of root obstacle avoidance needs the polar transport of auxin to occur [16]. Jasmonic acid (JA) and salicylic acid (SA) contents are also changed when roots are subjected to mechanical impedance [17]. Gibberellin (GA) negatively regulates the radial growth of roots by modulating the development of the apical meristem [18]. Multiple hormone signals also show complex crosstalk in affecting root growth. Huang et al. [19] found that ETH accumulation in the rice rhizosphere could promote ABA and auxin synthesis to achieve radial growth. Furthermore, ETH could mediate lateral root development by enhancing auxin signaling [5] and controlling the deferred elongation of elongation-zone cells [20]. ABA can inhibit root growth by delaying cell elongation and interacting with related signals, such as cytokinins (CKs), auxin, ETH, active oxygen (ROS), Ca^2+^, and so on [21,22,23]. These findings highlight the roles of plant hormones in root growth regulation. The relationship between the response to rhizosphere pressure in plant roots and specific hormones’ regulation needs to be further explored.

Arrowhead (*Sagittaria trifolia* L.) is a flowering wetland perennial herb of the Alismataceae family and also is an aquatic vegetable. The tips of the underground stolons of arrowhead grown in paddy fields can expand to form corms, the edible organs (underground storage organs) of arrowheads. Arrowhead corms contain abundant nutrients, such as protein, amino acids, and so on, as well as being rich in vitamins, therefore presenting high market value and being well loved by consumers [24]. At present, some problems have existed in arrowhead production that have profoundly affected the sustainable development of arrowheads. Corm harvesting is relatively difficult and cannot be mechanized since arrowheads are grown in paddy fields, and the labor force is still scarce. Low-efficient fertilizer utilization has also led to the abuse of resources and limited the scale of production. This is not uncommon in aquatic vegetable crop production [25]. Hydroponics is an effective way to address these matters as well as produce high-quality and residue-free vegetables [26]. No attempt has been made to cultivate arrowheads using hydroponics until now. Hence, we proposed a strategy to produce arrowheads via nutrient solution in order to promote the green and sustainable development of arrowheads.

In this study, we found that the corms of arrowheads did not expand over time under hydroponic conditions compared with those in sand-cultured conditions. It is speculated that this phenomenon may be caused by the decreased rhizosphere pressure in hydroponic conditions. In order to explore the potential mechanism of rhizosphere-pressure-induced corm expansion in arrowheads, a comparative analysis between sand-cultured and hydroponic treatments of arrowheads was performed, including transcriptome analysis, endogenous hormone determination, and related gene expression detection. This report provides new insight for exploring the effect of rhizosphere pressure on the formation and expansion of arrowhead corms and also lays a genetic foundation for directing arrowhead hydroponics. It gives the reference significance for subsequent research on rhizosphere pressure and the enlargement of underground storage organs.

## 2. Results

### 2.1. Plant Growth Analysis

**Figure 1:**
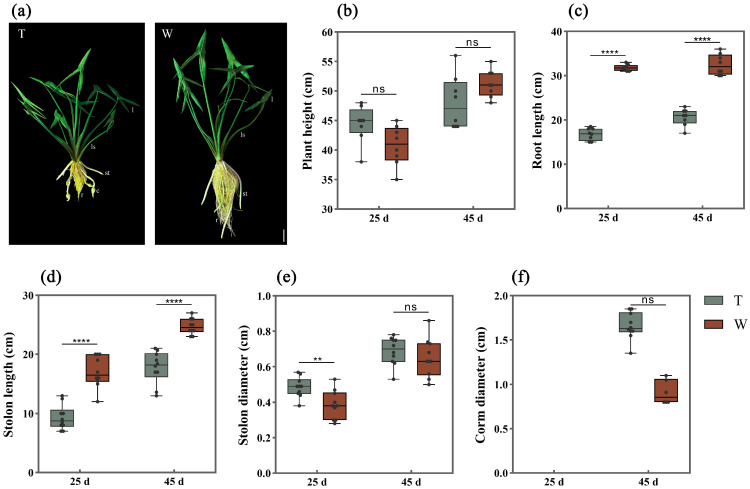
Morphological comparison of arrowheads in contrasting conditions. (**a**) Plant growth status of arrowheads 45 d after planting. l: leaf, ls: leafstalk, st: stolon, r: root, and c: corm. Bar represents 10 cm. (**b**–**f**) Plant height, root length, stolon length, stolon diameter, and corm diameter of two treatments 25 d and 45 d after planting. T and W represent sand culture and hydroponics, respectively. The error line indicates the standard deviation (SD) of multiple repeated samples. * means the significance of the difference between two treatments with Student’s *t*-test, ns: no significance, ** *p* < 0.01, **** *p* < 0.0001.

Comparing the underground growth of arrowheads between the sand culture and hydroponics, we found that the corms expansion of arrowheads in hydroponics was significantly delayed than that in sand culture. The arrowhead corms cultured in sand began to expand 45 d after planting (Figure 1a). It could be seen that there was no significant difference in the average plant height of arrowheads between the two treatments (Figure 1b). The lengths of arrowhead roots and stolons in sand culture conditions were significantly shorter compared with those in hydroponics, indicating that rhizosphere pressure restricted the elongation of underground roots and stolons (Figure 1c,d). Stolons began to emerge approximately 25 d after planting and reached the initial stage of corms expansion 45 d after planting. The stolon apical parts of sand-cultured arrowheads began to expand into corms, whereas corm expansion in stolon apical parts from hydroponics arrowheads was almost undetectable (Figure 1f).

### 2.2. Anatomical Analysis

**Figure 2:**
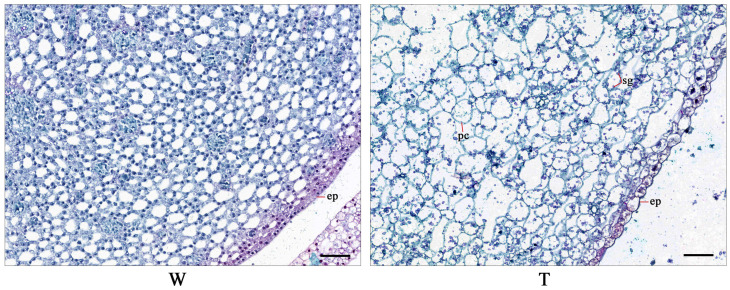
Anatomical structures of expanded and unexpanded arrowhead corm parts. Sampling time was 45 d after planting. T and W represent sand culture and hydroponics, respectively. ep: epidermis, pc: parenchyma cell, sg: starch granule; bar represents 100 μm.

In order to further visualize the histological characterization of corm expansion, paraffin-embedded tissue sectioning was conducted. The cross-sections of the corm formation parts in the initial expansion stage under the two conditions are shown in Figure 2. During the corm formation under sand culture conditions, parenchyma cells were significantly expanded in size, whereas the cell size in the unexpanded stolon tips in hydroponic conditions was small. Compared with those under hydroponic treatment, the corm cells in sand culture were larger based on the statistical cell length and width data (Table S4). In expanded corms from sand culture conditions, more starch granules were observed, but not in hydroponic arrowheads. The above results indicate that the formation of arrowhead corms in sand culture conditions derived from the increased cell size as well as being accompanied by starch accumulation.

### 2.3. DEGs Identification and Functional Annotation

**Figure 3:**
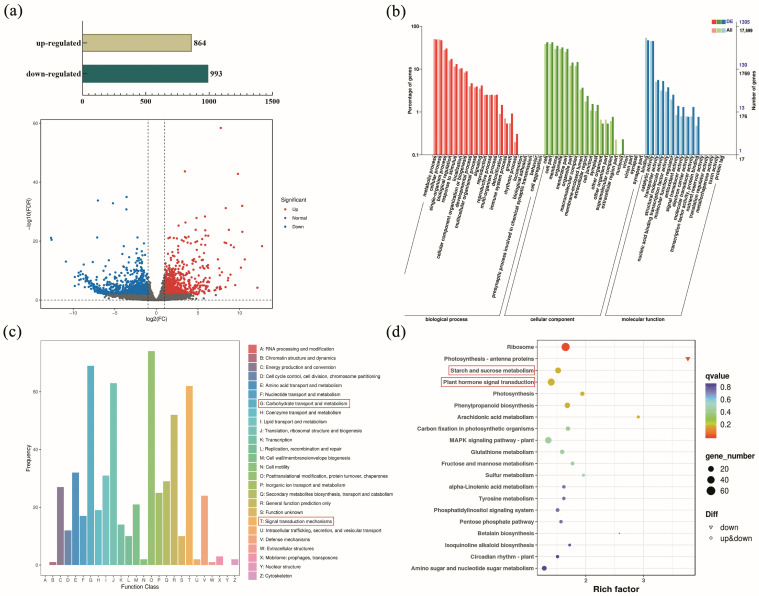
Transcriptome identification. (**a**) Volcano map and quantity statistics of DEGs. (**b**) GO annotation of DEGs. (**c**) COG functional classification of DEGs. (**d**) Top 20 KEGG metabolic pathways of DEGs. The pathway circled in red box indicates that it is a significantly enriched pathway that we noticed.

The transcriptome data on samples from the sand culture and hydroponics groups were employed for comparative analysis, and FDR < 0.01 and FC ≥ 2 were used as the screening criteria, yielding a total of 1857 DEGs. A total of 864 and 993 genes were upregulated and downregulated, respectively (Figure 3a). DEGs were clustered, annotated, and enriched. All the functional annotations of DEGs are shown in Figure 3b–d. A total of 1305 DEGs were annotated through GO enrichment and divided into three categories: biological processes, cellular components, and molecular functions (Figure 3b). For cellular components, approximately 42.38% of DEGs were clustered in the cell and cell parts, which account for the highest proportion, followed by 34.87% of DEGs in membranes, organelles, and macromolecular complexes. In terms of molecular function, the highest proportion of DEGs was annotated as binding, followed by catalytic activity, transporter activity, structural molecule activity, and nucleic-acid-binding transcription factor activity, suggesting the DEGs may have widely participated in the synthesis and transport of intracellular substances. For biological processes, metabolic processes enriching 49.04% of DEGs showed the highest proportion, followed by cellular processes and single-organism processes, implying that the DEGs between samples from hydroponics and sand culture were involved in cell metabolism, growth, and signaling.

Based on the COG functional classification of DEGs, a total of 602 DEGs were annotated into 25 functional categories (Figure 3c). Among the DEGs associated with post-translation modification, protein update and chaperones accounted for the highest proportion of 12.29%, followed by carbohydrate transport and metabolism (11.46%), translation, ribosomal structure, and biogenesis (10.47%), and signal transduction mechanisms (10.30%). In addition to basic molecular functions and essential life activities, carbohydrate transport and metabolism may play an important role in the formation of arrowhead corms. The function of proteins related to the signal transduction mechanism in corms is also worthy of attention.

An assay of KEGG pathway enrichment was performed. A total of 587 DEGs were annotated into fifty KEGG metabolic pathways with five categories: cellular process, environmental information processing, genetic information processing, metabolism, and organic systems. The top 20 pathways estimated by KEGG enrichment degree are listed in Figure 3d. Notably, numerous DEGs are annotated as plant hormone signal transduction pathways, suggesting that hormone signaling was critical for corms expansion. A range of genes involved in starch and sucrose metabolism were also identified, indicating that the corm formation was accompanied by starch accumulation, which is consistent with our observed anatomical results.

### 2.4. Hormone Level Determination

**Figure 4:**
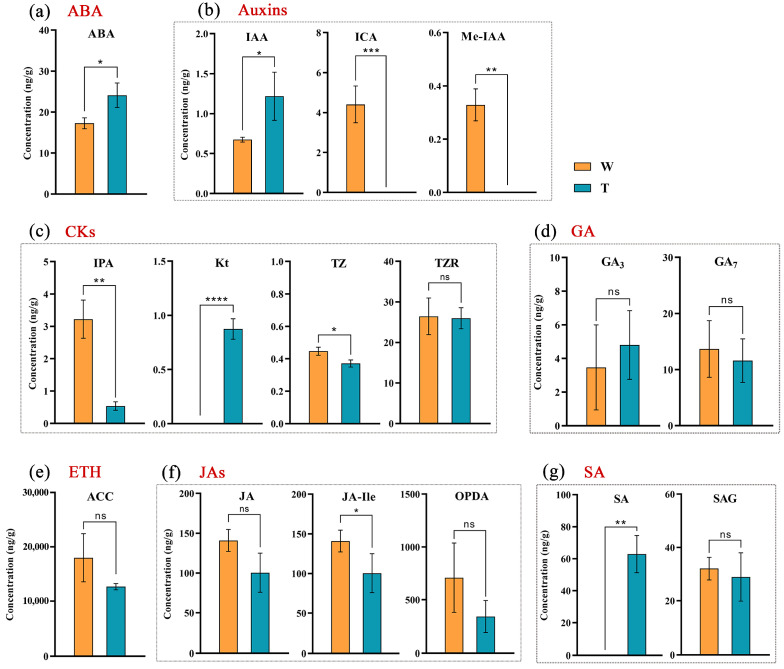
Contents of hormones in two treatments at the initial stage of corm expansion. (**a**–**g**) Hormone contents of ABA (**a**), auxins (**b**), CKs (**c**), GA (**d**), ETH (**e**), JAs (**f**), and SA (**g**) in corms in contrasting conditions. The dashed boxes indicate the types covered by each hormone. T and W represent sand culture and hydroponics, respectively. Column heights indicate hormone contents. The error line indicates the standard deviation (SD) of three repeated samples. * means the significance of the difference between two treatments analyzed with Student’s *t*-test, ns: no significance, * *p* < 0.05, ** *p* < 0.01, *** *p* < 0.001, **** *p* < 0.0001.

Based on the results of the transcriptome analysis, our follow-up research mainly focused on the role of hormone signaling in corm formation. The contents of seven endogenous hormones, ABA, auxins, CKs, GA, JAs, and the ethylene precursor ACC (l-aminocyclopropane-l-carboxylic acid), in expanded corms and unexpanded stolon tips from sand-cultured and hydroponic arrowheads at 45 d after planting were detected (Figure 4). The content of ABA in sand-cultured arrowhead corms was remarkably higher than that in hydroponics samples. The contents of two types of auxins, ICA and Me-IAA, were higher in hydroponics samples than those in sand culture samples. Accumulations of GA_3_ and GA_7_ differed between sand culture and hydroponics samples. The ACC content in both treatment groups was high, and no significant difference was found. The levels of JA were similar in the two treatments and were high overall. More OPDA (12-oxophytodienoic acid), the precursor for JA synthesis, was determined in the samples from hydroponic treatments. High-level salicylic acid (SA) accumulation was detected in sand-cultured corms and not in hydroponic samples. Taken together, the corms expansion induced by rhizosphere pressure seems to be linked to the increased accumulations of ABA and SA. Furthermore, JA may also participate in the process of corm expansion. The high ACC content suggested a considerable amount of ETH existed in the arrowhead rhizosphere.

### 2.5. Expression Profiles of Hormone-Related Genes in RNA-Seq

**Figure 5:**
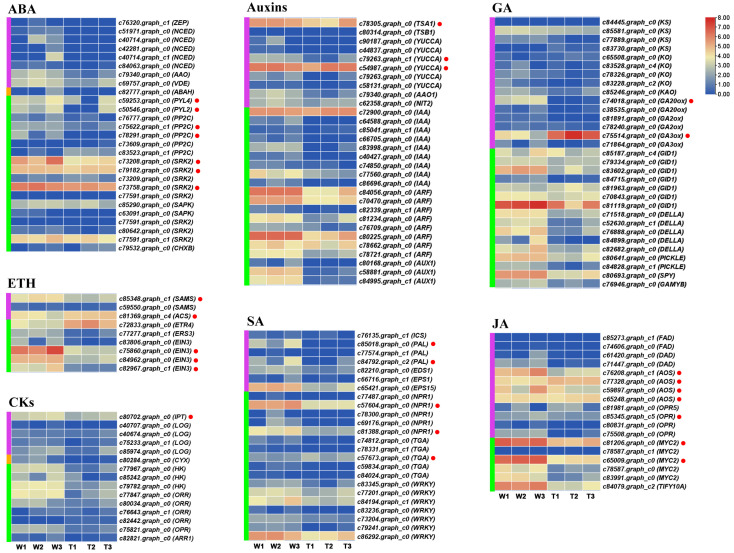
Expression profiles of genes related to plant hormone metabolism in sand culture and hydroponics. Green, orange, and purple vertical lines indicate biosynthesis, degradation, and signaling genes, respectively (color figure online). The gene with a red dot behind it indicates that it is a differentially expressed gene (DEG). W1–3 and T1–3 represent three biological repeats in hydroponics and sand culture, respectively.

In order to further explore the molecular regulatory mechanisms of differences in hormone contents, the expression profiles of genes related to the metabolism of seven hormones (ABA, auxins, GA, ETH, CKs, SA, and JA) were analyzed based on RNA-Seq data (Figure 5). The genes involved in multiple hormones metabolism showed significant or non-significant transcription differences. For ABA metabolism, most genes related to ABA synthesis were highly expressed in hydroponics, and the expression of the *StPYL* (encoding ABA receptor protein synthesis) and *StPP2C* (encoding 2C type protein phosphatases) families were also upregulated in hydroponics. The *StSRK2* family, the homologous genes of SnRK/SRK (serine/threonine-protein kinase), was also highly expressed in hydroponics. In auxins metabolism, several auxin-synthesis-related genes showed high transcription levels, such as *StTSA1* and *StYUCCA*. The genes encoding *StIAA* (auxin-responsive protein IAA), *StARF* (auxin response factor), and many homologous genes were highly expressed in the two treatments. *StGA3ox1* catalyzing active GA synthesis was significantly upregulated in sand-cultured corms. In addition, the genes encoding StGIDs and StDELLAs were involved in GA synthesis regulation and showed relatively stable expression in the corm samples. *StACS* regulating ETH biosynthesis exhibited high transcription levels in both arrowhead samples, whereas the expression levels of genes involved in ETH signal transduction in hydroponic samples were upregulated compared with sand-cultured corms. Genes involved in SA signaling were upregulated in hydroponics, such as the genes encoding the StNPR1 receptor family and the StWRKY transcription factor. *StMYC2* family genes, the main regulators of the JA signal transduction pathway, most of all exhibited high expression in hydroponics, and the expression levels of genes (*StAOS* and *StOPRs*) related to JA biosynthesis in hydroponics were higher than those in sand culture.

### 2.6. Verification of Differentially Expressed Genes in Hormone Signaling

**Figure 6:**
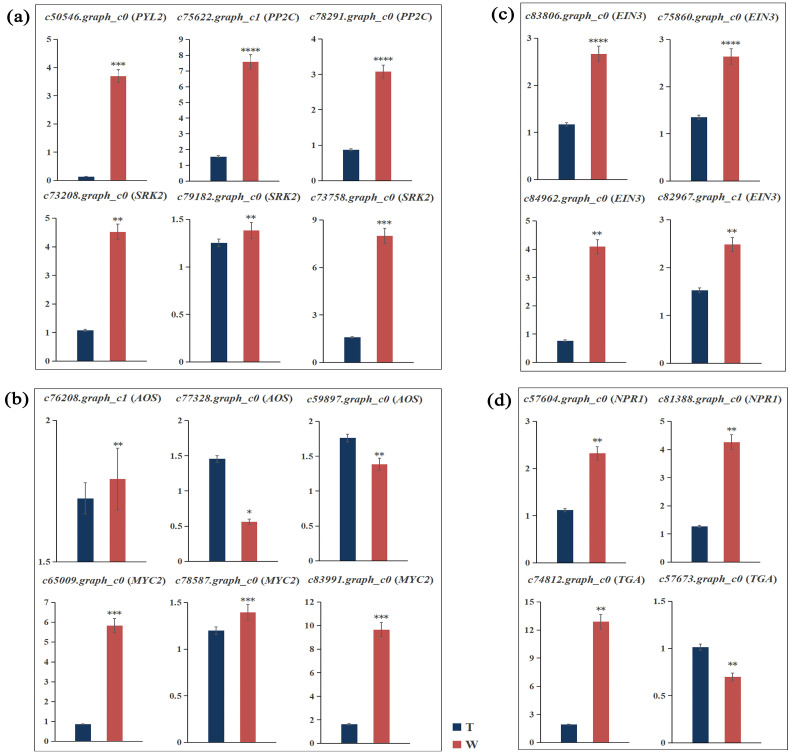
Relative expressions of ABA, JA, ETH, and SA metabolism-related genes in samples from sand culture and hydroponics. (**a**–**d**) Relative expressions of genes related to ABA (**a**), JA (**b**), ETH (**c**), and SA (**d**). Column height indicates gene expression level. T and W represent sand culture and hydroponics, respectively. The error line indicates the standard deviation (SD) of three replicates. * means the significance of the difference between two treatments analyzed with Student’s *t*-test, * *p* < 0.05, ** *p* < 0.01, *** *p* < 0.001, and **** *p* < 0.0001.

To further verify the RNA-Seq results, the relative expression of genes participating in ABA, JA, ETH, and SA metabolism was quantified via RT-qPCR assay. Some genes in the PYL-PP2C-SnRK2 pathway, closely associated with the ABA signaling pathway, were identified from transcriptome data. The RT-qPCR results show that the expression levels of these genes in hydroponics were higher than those in sand culture, which is consistent with the results analyzed using RNA-Seq (Figure 6a). The expression levels of genes encoding allene oxide synthase (AOS) and MYC2 transcription factor in the JA synthesis pathway were verified. The results show that the expression of *StMYC2* genes almost matched the expression profiles analyzed with transcriptome, and the expressions of two *StAOS* genes were downregulated in the samples from hydroponics (Figure 6b). The expression levels of four ethylene insensitive 3 (EIN3) family genes were upregulated in hydroponics (Figure 6c) and shared a similar profile to the RNA-Seq data and ACC content. We also detected the expressions of the *StNPR1* and *StTGA* transcription factor genes encoding the SA receptor protein (Figure 6d). *StNPR1* was upregulated in hydroponics samples, while *StTGAs* were upregulated or downregulated, which may be related to the differential expression of TGA family proteins. The expression changes in hormone-related genes contributed to the differential accumulation of endogenous hormones in arrowhead corms.

## 3. Discussion

When the rhizosphere pressure from the soil increases, most plants avoid injury by reducing root elongation, increasing the root cross-sectional area, or bending so as to enhance their adaptability to soil resistance [1]. Numerous studies have reported the negative effects of rhizosphere pressure on underground root growth [3,9]. Positive effects of rhizosphere pressure such as increased biomass have also been reported [4,27]. The role of rhizosphere pressure in the development of underground storage organs has not received much attention at present. Crops with underground roots or stems as storage organs, such as potatoes and sweet potatoes, also need the induction of resistance in the soil to achieve a high yield during production [28,29,30]. The rhizosphere pressure exerted on the underground part of plants in sand culture (~1.78 g/cm^3^) was higher than that in hydroponics (~1.00 g/cm^3^). Therefore, we speculated that rhizosphere pressure promoted the expansion of underground storage organs in crops. In this study, the effect of rhizosphere pressure on the arrowhead corms expansion in hydroponics and sand culture (simulated soil cultivation) was observed. It was found that the corm formation was significantly delayed under hydroponics conditions compared with sand culture. At 45 d after planting, the cells in the corm formation parts were expanded and accompanied by starch accumulation, which is consistent with the anatomic structural changes related to storage organ expansion described in previous studies [31]. In contrast, the cells in the corm expansion parts of hydroponic arrowheads were small and closely arranged, resulting in no expanded corm being generated. Based on these results, we concluded that rhizosphere pressure was the key factor that induced corms expansion in arrowheads.

What responds to rhizosphere pressure and regulates corms expansion in arrowheads? To preliminary explain this point, we carried out transcriptome sequencing using the expanded corms and unexpanded stolon tips from sand culture and hydroponics. A number of DEGs were identified and annotated through the GO, COG, and KEGG databases. In COG classification, the majority of DEGs were annotated into two categories: carbohydrate transport and metabolism and signal transduction mechanism. The KEGG analysis showed that the DEGs involved in plant hormone signal transduction accounted for the highest proportion, indicating that plant hormones may be vital regulators in modulating arrowhead corm expansion induced by rhizosphere pressure. It is well known that hormones are important chemical messengers in plants that regulate plant growth and development and participate in external environmental responses and transmit certain signals [32,33]. We speculated that rhizosphere pressure regulates the morphological changes in the stolon tip by altering plant hormone accumulation and signal transduction [34,35], thus leading to the formation of corms in sand-cultured arrowheads.

It has been reported that the regulation by rhizosphere pressure of plant root growth involves a variety of hormones, including ETH, auxins, and ABA [14,36]. Under high rhizosphere pressure (soil compaction), a large amount of non-diffusible ETH accumulation activates ABA and auxin signals, leading to changes in root morphology [36,37]. The presence of a water layer also inhibits the diffusion of the gas hormone, ETH, in both hydroponic and sand-cultured arrowheads, resulting in a high accumulation of the ETH precursor ACC [38,39]. ABA is a plant hormone that plays an important role in resisting abiotic stresses [40], and its role in inducing the radial thickening of roots has also been reported [19]. In our work, the ABA content in the arrowhead corms under sand culture conditions was significantly increased, implying that ABA may respond to rhizosphere pressure to promote corms expansion. The auxin content also varied in the arrowhead samples of the two treatment groups and may be regulated by ETH and ABA [41]. In addition, several types of JAs showed differential accumulation in the arrowhead samples from the two treatment groups. When plants were stressed, JA increased and interacted with other hormones, and inhibiting root elongation was also a typical trait [42]. Therefore, we speculated that JA may be involved in the rhizosphere pressure response in the underground parts, which provides new evidence of JA signals mediating rhizosphere pressure to change rhizosphere characteristics. SA has been reported to be associated with plants’ sense of stress [43,44], which could be detected only in expanded corms from sand culture, implying its potential function in facilitating arrowhead corms or interacting with other hormones. Meanwhile, the specific relationship between JA/SA and rhizosphere pressure is still unclear.

In plants, the accumulation of hormones is usually linked to the expression of genes in biosynthesis, degradation, and signal transduction pathways [32]. To further explore the regulation mechanisms of hormones, we retrieved the hormone-metabolism-related genes and verified the expression levels. ABA was highly accumulated in the expanded arrowhead corms from sand culture, and the expression of genes involved in ABA signal transduction was more active in hydroponics, and we speculated that the gene’s expression may be influenced by the feedback regulation of ABA content, and the increased ABA level inhibits the transcription activities of *PP2C* and *SRK2* [15,45]. In hydroponic arrowheads, two highly expressed *AOS* genes might contribute to the increases in JA and OPDA contents [46,47]. EIN3 exerted a decisive role in the ETH signaling pathway, and it could mediate the interaction between JA and ETH [48]. The genes encoding EIN3 proteins showed high expression in hydroponic arrowhead samples, indicating that ETH signaling was activated in hydroponics conditions. The *PAL* genes, *c85018.graph_c0* and *c84792.graph_c2*, in the upstream of SA biosynthesis were upregulated in hydroponics, which was contrary to the results of SA accumulation. A possible reason is that the synthesis of SA underwent multiple catalytic steps, and the intermediate products (such as *trans*-cinnamic acid and shikimic acid) produced during this process were also involved in the synthesis of other secondary metabolites; thus, the expression of upstream genes did not show a direct impact on the accumulation of SA [49,50]. Another interpretation could be that the hormones’ biosynthesis also depended on the post-transcriptional control of genes and the regulation of protein levels [51].

**Figure 7:**
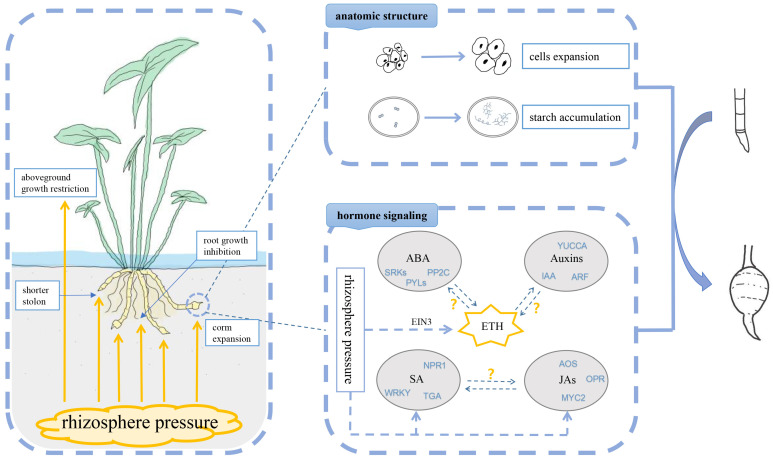
Model diagram of rhizosphere pressure response of arrowheads. In ‘hormone signaling’ the ‘?’ means that the interaction mechanism may exist, but not yet clear.

Previous studies have not mentioned the relationship between the corm expansion in underground stolon tips and rhizosphere pressure in arrowheads [52,53]. Herein, we present a model diagram to explain the potential mechanism (Figure 7). The effects of the rhizosphere pressure provided by the sand on the growth of arrowheads are briefly summarized. The mechanism of corms expansion was preliminarily demonstrated via anatomical structure observation and hormone signaling analysis.

## 4. Materials and Methods

### 4.1. Plant Materials and Cultivation Conditions

In this experiment, 20 cm high seedlings of *S. trifolia.* cv. ‘Baimati’ (BMT) were planted in 38 × 25 cm nonporous plastic pots. The plants were divided into two groups to perform the hydroponics and sand-cultured treatments. For the sand-cultured treatment, coarse river sand was used to simulate soil pressure, and a self-developed nutrient solution was added to rise 4 cm above the surface of the sand (Table S1). The same nutrient solution was applied for the hydroponic treatment, and the plants were fixed with a black foam plate with a hole in the center to avoid underground light. Herein, we define the gravity density (weight per unit volume of cultivated medium) to describe the rhizosphere pressure in the two treatments. The gravity density was approximately 1.00 g/cm^3^ and 1.78 g/cm^3^ in the hydroponics and sand cultures, respectively. The nutrient solution was supplemented every five days. The growing season was from April to July, and the average temperatures were 27 °C/day and 18 °C/night.

In this study, the growth of arrowheads after planting was divided into three stages: the vegetative growth stage, the stolon development stage (which began 25 d after planting), and the corm expansion stage (which began at 45 d after planting). At 25 d and 45 d, the plant height (an indicator of the above-ground part), root length, and stolon diameters (indicators of the underground growth) were measured using graduated scales and a vernier caliper. At least 10 plants were measured for each indicator. The formation and expansion of corms in the two treatment conditions were observed and recorded. The samples were harvested at the initial stage of corms expansion in the sand culture (45 d after planting) and then stored at −80 °C for subsequent experiments. At least three biological replicates were set for each treatment.

### 4.2. Observation of Anatomical Structure of Corms

Expanded arrowhead corms in the sand culture and unexpanded stolon apical parts in the hydroponics culture were sampled for paraffin cross-sections to observe the differences in anatomical structure. Firstly, the collected samples were fixed with FAA stationary liquid (5 mL of formalin, 5 mL of glacial acetic acid, and 90 mL of 70% alcohol). Then, the corm tissues were dehydrated and embedded in paraffin. Treated tissues were sliced with HistoCore BIOCUT (Leica, Wetzlar, Germany) and stained with plant saffron fast green dye prepared by FreeThinking (Nanjing FreeThinking Biotechnology Co., Ltd., Nanjing, China). The obtained tissue sections were observed using an Olympus CX23 microscope (Olympus, Tokyo, Japan) and scanned using XZ Sunny HS6 (Sunny Optical Technology (Group) Co., Ltd., Ningbo, China).

### 4.3. RNA Extraction and Detection

According to the operating instructions, total RNA was extracted from corm samples with the RNAprep Pure Plant Kit (Proteinssci, Shanghai, China). The concentration of extracted RNA was detected using Nanodrop^2000^ (Thermo Scientific, Wilmington, DE, USA), and the integrity of the RNA was tested using Agient2100 and LabChip GX (PerkinElmer, Waltham, MA, USA). For the evaluation of RNA quality, RNA samples were separated via 1.0% agarose gel electrophoresis, showing that the quality of the RNA was good enough for further analysis (Figure S1).

### 4.4. Transcriptome Sequencing, Assembly, and Functional Annotation

The VAHTS Universal V6 RNA-seq Library Prep Kit for Illumina^®^ was used for the synthesis of cDNA and construction of the final cDNA library. The sequencing of library products was performed with the Illumina NovaSeq high-throughput sequencing platform (Illumina, San Diego, CA, USA). The generated data were assembled using Trinity (v2.5.1) [54] software. A total of 44.65 Gb of clean data was obtained from six arrowhead samples. The generated data per sample reached 6.60 Gb. The percentage of Q30 bases was more than 94.78%. In total, 50,031 unigenes were obtained after assembly, among which 15,728 unigenes exceeded 1 kb in length (Table S2). The N50 number of unigenes was 1723 with an average length of 2287 bp (Table S3). Unigene sequences with a length greater than 500 bp accounted for 54.32% (Figure S2). Taken together, the assembly was high-quality, and the results could be used for further analysis. DIAMOND (v2.0.4) [55] software was used to blast the unigene sequences with the NR, Swiss-Prot, GO, COG, KOG, eggNOG, and KEGG databases and then, the annotation information on the unigenes was obtained (Table 1).

Bowtie [56] was used to compare the sequenced reads with the unigene library. According to the comparison results, the expression abundance of identified unigenes was estimated with RSEM [57] and expressed as FPKM (fragments per kilobase million) values. Differential expression analysis was conducted with the following screening criteria: FDR (false discovery rate) of <0.01 and FC (fold change) of ≥2.

### 4.5. Endogenous Hormones Determination

The samples, including expanded corms and unexpanded stolons apical parts, were harvested from the sand culture and hydroponics at the initial stage of corms expansion. Each sample after grinding was added into a 2 mL brown centrifuge tube containing 1 mL of methanol and a mixed internal standard stock solution. The mixture was subjected to ultrasonic extraction for 10 min and then transferred to a metal bath for 4 h. After centrifugation for 10 min under 12,000 rpm/4 °C conditions, the supernatant was collected. The remaining residue was resuspended using 0.5 mL of methanol and continued undergoing oscillating extraction for 2 h in a metal bath. The supernatants obtained from two extractions were mixed, centrifuged, and then filtered through a 0.22 μm filter membrane. The filtered samples were loaded into a high-performance liquid chromatography–electrospray ionization mass spectrometry (LC-MS) system [58] to estimate the levels of seven kinds of endogenous hormones (ABA, auxins, CKs, GA, ETH, JAs, and SA).

### 4.6. Identification and Analysis of Genes Involved in Various Hormones’ Metabolism Based on RNA-Seq Data

Genes involved in the biosynthesis, degradation, and signal transduction of plant hormones (auxins, CKs, GA, ABA, ETH, SA, and JA) were retrieved from the transcriptome database via sequence homology blast. The heatmap of gene expression levels was drawn using TBtools (v1.120) software, in which the expression per gene was quantified using log_2_ (FPKM + 1).

### 4.7. Gene Expression Analysis Using RT-qPCR

In order to further verify the expression results analyzed with the RNA-seq data, the expression levels of 20 target genes in the samples from the sand culture and hydroponics were detected using real-time quantitative PCR (RT-qPCR) with a CFX96 system (Bio-Rad, Hercules, CA, USA). *SLEEPER* was used as the internal reference gene, and the specific primers for RT-qPCR detection were designed using Primer 6.0 and are listed in Table 2. The specificities of the primers were determined with melting curves (Figure S3). The reaction volume was 10 μL, which contained 4 μL of diluted cDNA, 1 μL of a forward or reverse primer, and 5 μL of the iTaq Universal SYBR Green Supermix Kit (Bio-Rad, Hercules, CA, USA). The reaction steps were as follows: pre-denaturation at 95 °C for 3 min, 95 °C for 10 s, and 60 °C for 5 s, and a total of 39 cyclic reactions. Three technical repetitions were set for each reaction. The relative expression value was calculated using the 2^−ΔΔCt^ method [59].

### 4.8. Statistical Analysis

The means and standard deviation (SD) of the data were calculated using Excel 2019. The difference significance between treatments was analyzed using a *t*-test with GraphPad Prism 9 software.

## 5. Conclusions

The corms expansion of arrowheads is affected by complex regulatory factors involving the signal transduction of various hormones. In this study, we compared the expansion of arrowhead corms under hydroponics (with lower rhizosphere pressure) and sand-culture (with higher rhizosphere pressure) conditions, finding that rhizosphere pressure could induce the formation of arrowhead corms. The endogenous hormones, ABA, JA, ETH, and SA, may respond to rhizosphere pressure, thus regulating corms expansion in sand-cultured arrowheads. Through the observation of anatomical structure, we found that the expansion of corms was inseparable from starch accumulation. Our current works will provide a meaningful reference for further understanding the corm expansion mechanism and exploring the influence of rhizosphere pressure/mechanical impedance on the formation of plant underground storage organs.

## Figures and Tables

**Table 1:** Unigenes functional annotation.

Annotation Database	Number of Annotated Genes	Percentage of Annotated Genes (%)
COG Annotation	5926	11.85
GO Annotation	17,699	35.38
KEGG Annotation	13,816	27.62
KOG Annotation	11,765	23.52
Pfam Annotation	15,924	31.83
Swissprot Annotation	13,673	27.33
TrEMBL Annotation	21,016	42.01
eggNOG Annotation	18,223	36.42
Nr Annotation	21,053	42.08
All Annotated	22,050	44.07

**Table 2:** The specific primers used for RT-qPCR assay.

Gene ID	Primer Sequences (5′→3′)	
	Forward	Reverse
*c50546.graph_c0* (*PYL2*)	GTCTGGTCCATCGTCCGTAGCT	TTGGTGTCCTCCTCGCTGTTCC
*c75622.graph_c1* (*PP2C*)	TCCCTGAGCCAGAGGTGAGATT	TGGAGTGCAAGCCTGGATAGGT
*c78291.graph_c0* (*PP2C*)	CCAACCAGGACTCCGCCATACT	CGGTCATTGCCTTGGTCGTCAT
*c73208.graph_c0* (*SRK2*)	GCATTCACAGCCTAAGTCAACAGTT	CCGCAGAGATGGATTGCCAACAA
*c79182.graph_c0* (*SRK2*)	CCGCTTCTTCTTCCAGCAACTC	CACACGACCATACATCCGCAAT
*c73758.graph_c0* (*SRK2*)	ACTCCAAGTCCTCCGTGCTTCA	CAGACTGGGCTGCTTCGGTTAG
*c76208.graph_c1* (*AOS*)	CCTCTTCGCCACCTGCTTCAAC	GCCTCGTAGACCACCGACTTCA
*c77328.graph_c0* (*AOS*)	AGGACCGCCTTGACTACTTCTACC	TTGACGGCATGTAGGTTCCTGTGA
*c59897.graph_c0* (*AOS*)	ATCGAGGAGCAGATCGCCAAGG	TGAAGCCGCCGAAGGAGTTGA
*c65009.graph_c0* (*MYC2*)	AGGACCATATCATCGCCGAGAGG	ACTTCTTAACCAGGACAACCGACTC
*c78587.graph_c0* (*MYC2*)	CCAACCTGCACAACAACCTCTG	AAGCGGCGTCTTCGTGTAGC
*c83991.graph_c0* (*MYC2*)	TCACGCTCCCTACTGGCAAAGA	ACCCTCACTTGGCGGACTATCA
*c83806.graph_c0* (*EIN3*)	CATCCACCACCGCCTCAATCCT	CTGCTGCTGCCGTTGCTGTT
*c75860.graph_c0* (*EIN3*)	GCAGCAGCAGCAGTTCAATCC	CGGAAGTCACCAGCGGCATT
*c84962.graph_c0* (*EIN3*)	CGAACCTCAAGTGCGTGTCCTT	GCGTTGCCCATCACCCAGAA
*c82967.graph_c1* (*EIN3*)	AGCACAGTGGTGAGAAGCAGGA	AACTTCCGTTGTCGCTCCCTCT
*c57604.graph_c0* (*NPR1*)	TGCGAGAAGTTGCTGGACAAGT	GCGTATGCGTCATCCAAGGTTG
*c81388.graph_c0* (*NPR1*)	TGGCAGCGGTGTACTGGTCAA	ACGGTGAGAGGTGAGCATGGAA
*c74812.graph_c0* (*TGA*)	CGCTGCTTCATGTGGCTTGGT	CGGTTGTGGTACTCGCCTATGG
*c57673.graph_c0* (*TGA*)	CGCCAGAGAATGAGGTGAGGTT	AGTCCTTGCGAGAGTGAATCCT
*c76930.graph_c0* (*SLEEPER*)	CCTGTATTGCTTGGAAGTGGTAT	CCGTCGTCTTGATTGAATGGAT

## Data Availability

All relevant data can be found within this article and its Supporting Materials.

## Supplementary Materials

The supporting information can be downloaded at: https://www.mdpi.com/article/10.3390/ijms241512345/s1.
