# Supplementary material for: The Roles of Hormone Signals Involved in Rhizosphere Pressure Response Induce Corm Expansion in Sagittaria trifolia
Source: Int J Mol Sci. 2023 Aug 2;24(15):12345. doi: 10.3390/ijms241512345 (PMC10419225; doi:10.3390/ijms241512345)
Supplement: Supplementary file 1 [file ijms-24-12345-s001.zip › ijms-2466788-supplementary.pdf]

The roles of hormone signals involved in rhizosphere pressure response induces corm expansion in *Sagittaria trifolia*

Enjiao Li[a], Jing Tang[a], Jiexia Liu[a], Zhiping Zhang[a], Bing Hua[a], Jiezeng Jiang[a], Minmin Miao[a,b,c]*

[a]College of Horticulture and Landscape Architecture, Yangzhou University, Yangzhou 225009, China

[b]Joint International Research Laboratory of Agriculture and Agri-Product Safety of Ministry of Education of China, Yangzhou University, Yangzhou 225009, China

[c]Key Laboratory of Plant Functional Genomics of the Ministry of Education, Yangzhou University, Yangzhou 225009, China

***Corresponding author:** Minmin Miao

Telephone: +86-0514-87979344;

Fax: +86-0514-87347537;

Email: mmmiao@yzu.edu.cn.

The author responsible for distribution of materials integral to the findings presented in this article in accordance with the policy described in the Instructions for Authors (https://academic.oup.com/plcell/pages/General-Instructions) is: Minmin Miao (mmmiao@yzu.edu.cn).

Supplementary data

Table S1 Nutrient solution formula.

Macroelement	Vegetation growth （mg/L）	Reproductive growth （mg/L）	Microelement	Whole growth period （mg/L）
$Ca(NO_3)_2 \cdot 4H_2O$	1400	1700	KI	0.83
KNO_3	150	180	$MnSO_4$	22.3
KH_2PO_4	540	680	$ZnSO_4$	8.6
$MgSO_4 \cdot 7H_2O$	730	920	$Na_2MoO_4 \cdot 2H_2O$	0.25
K_2SO_4	260	320	$CuSO_4$	0.025
$Ca(HCO_3)_2$	110	140	$CoCL_2$	0.025
$(NH_4)_2CO_3$	80	100	$C_{10}H_{14}N_2Na_2O_8$	37.25
			$FeSO_4 \cdot 7H_2O$	27.85

Note: all used elements were water-soluble fertilizer.

Table S2 Statistics of sequencing data.

Treatment	Clean reads	Clean bases	Mapped reads	Q30 (%)	GC (%)
T1	26,742,777	7,968,124,396	21,084,167（78.84 %）	95.43	53.17
T2	28,630,932	8,559,962,478	23,935,910（83.60 %）	95.52	53.92
T3	25,317,454	7,571,165,656	20,783,819（82.09 %）	95.10	52.63
W1	24,515,010	7,338,799,168	20,719,486（84.52 %）	95.67	51.75
W2	22,056,195	6,602,091,438	18,509,493（83.92 %）	94.78	51.47
W3	22,078,177	6,605,229,418	18,571,522（84.12 %）	95.60	52.17

Table S3 Assembly results statistics.

Length Range	Transcript	Unigene
200-300	4807 (3.46 %)	3596 (7.19 %)
300-500	28,302 (20.37 %)	19,261 (38.50 %)
500-1000	28,251 (20.33 %)	11,446 (22.88 %)
1000-2000	38,356 (27.60 %)	8760 (17.51 %)
2000+	39,233 (28.24 %)	6968 (13.93 %)
Total Number	138,949	50,031
Total Length	213,736,062	50,699,114
N50 Length	2287	1723
Mean Length	1,538.23	1013.35

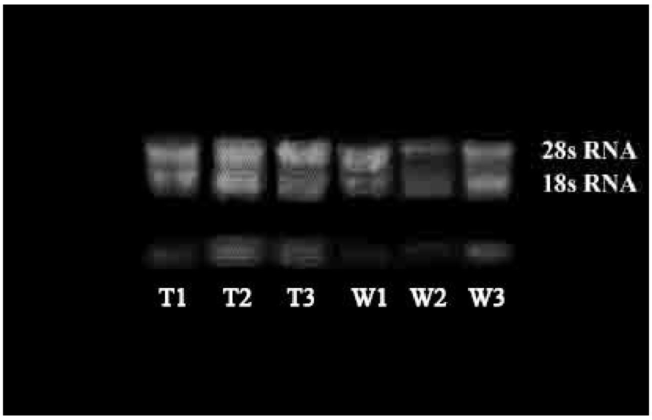

Figure S1 RNA quality detected by electrophoresis.

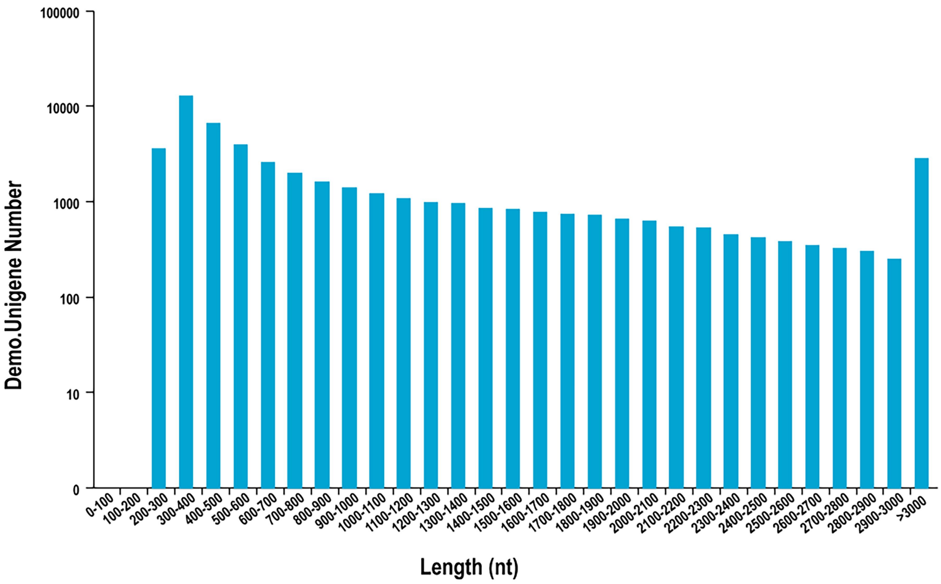

Figure S2 Unigenes length distribution.

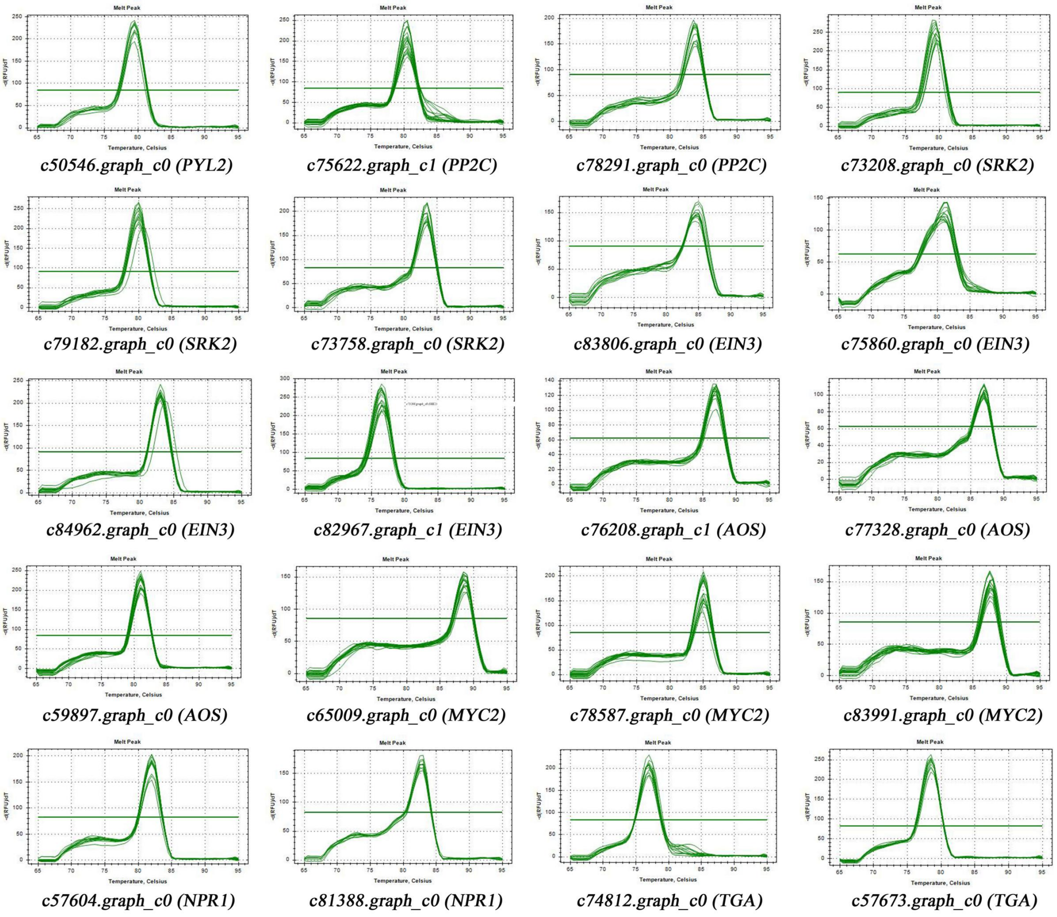

Figure S3 The melting curves of used primers for RT-qPCR detection.

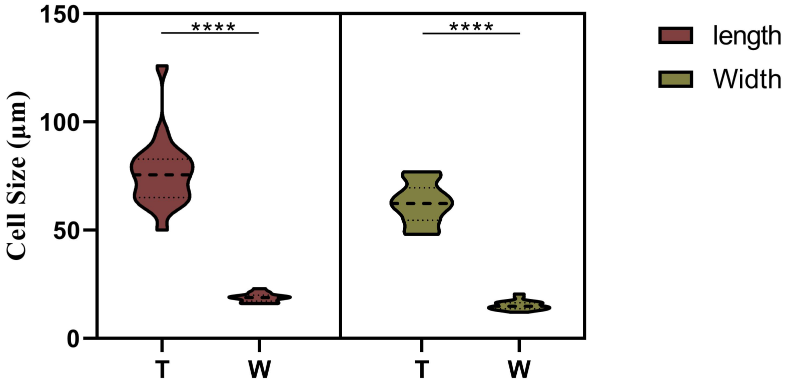

Figure S4 Measurement of cell size in two treatments.

20 cells were randomly selected from the same visual field, and the length and width were measured to estimate the size of the cells. T and W represent sand culture and

hydroponics, respectively. * means the significance of the difference between two treatments, **** $P < 0.0001$.
